# Novel Indel Variation of NPC1 Gene Associates With Risk of Sudden Cardiac Death

**DOI:** 10.3389/fgene.2022.869859

**Published:** 2022-04-11

**Authors:** Wenfeng Zhao, Qing Zhang, Jiawen Wang, Huan Yu, Xiaoyuan Zhen, Lijuan Li, Yan Qu, Yan He, Jianhua Zhang, Chengtao Li, Suhua Zhang, Bin Luo, Jiang Huang, Yuzhen Gao

**Affiliations:** ^1^ Department of Forensic Medicine, Medical College of Soochow University, Suzhou, China; ^2^ Institute of Forensic Medicine, Guizhou Medical University, Guiyang, China; ^3^ Department of Biological Science, Science School of Xi’an Jiaotong-Liverpool University, Suzhou, China; ^4^ Department of Epidemiology, Medical College of Soochow University, Suzhou, China; ^5^ Shanghai Key Laboratory of Forensic Medicine, Institute of Forensic Sciences, Ministry of Justice, Shanghai, China; ^6^ Faculty of Forensic Medicine, Zhongshan School of Medicine, Sun Yat-Sen University, Guangzhou, China

**Keywords:** sudden cardiac death, NPC1, indel polymorphism, rs150703258, genetic susceptibility

## Abstract

**Background and Aims:** Sudden cardiac death (SCD) was defined as an unexpected death from cardiac causes during a very short duration. It has been reported that Niemann-Pick type C1 (*NPC1*) gene mutations might be related to cardiovascular diseases. The purpose of the study is to investigate whether common genetic variants of *NPC1* is involved in SCD susceptibility.

**Methods:** Based on a candidate-gene-based approach and systematic screening strategy, this study analyzed an 8-bp insertion/deletion polymorphism (rs150703258) within downstream of *NPC1* for the association with SCD risk in Chinese populations using 158 SCD cases and 524 controls. The association of rs150703258 and SCD susceptibility was analyzed using logistic regression. Genotype-phenotype correlation analysis was performed using public database including 1000G, expression quantitative trait loci (eQTL), and further validated by human heart tissues using PCR. Dual-luciferase assay was used to explore the potential regulatory role of rs150703258. Gene expression profiling interactive analysis and transcription factors prediction were performed.

**Results:** Logistic regression analysis exhibited that the deletion allele of rs150703258 significantly increased the risk of SCD [odds ratio (OR) = 1.329; 95% confidence interval (95%CI):1.03–1.72; *p* = 0.0289]. Genotype-phenotype correlation analysis showed that the risk allele was significantly associated with higher expression of NPC1 at mRNA and protein expressions level in human heart tissues. eQTL analysis showed NPC1 and C18orf8 (an adjacent gene to NPC1) are both related to rs150703258 and have higher expression level in the samples with deletion allele. Dual-luciferase activity assays indicate a significant regulatory role for rs150703258. Gene expression profiling interactive analysis revealed that NPC1 and C18orf8 seemed to be co-regulated in human blood, arteries and heart tissues. *In silico* analysis showed that the rs150703258 deletion variant may create transcription factor binding sites. In addition, a rare 12-bp allele (4-bp longer than the insertion allele) of rs150703258 was discovered in the current cohort.

**Conclusion:** In summary, our study revealed that rs150703258 might contribute to SCD susceptibility by regulating NPC1 and C18orf8 expression. This indel may be a potential marker for risk stratification and molecular diagnosis of SCD. Validations in different ethnic groups with larger sample size and mechanism explorations are warranted to confirm our findings.

## 1 Introduction

Sudden cardiac death (SCD) refers to unexpected death due to cardiac causes within 1 h of symptom onset, regardless of the preexistence of heart disease (Zheng et al., 2001). It has been reported that SCD globally accounts for 4-5 million deaths per year (Chugh 2017) and is responsible for over the half of cardiovascular death (Deo and Albert 2012). In older age groups, SCD is often attributed to complications of coronary atherosclerosis, while in young people (<35 years of age) or people without structural heart disease, hereditary causes of death should be considered, such as channelopathies and cardiomyopathies (Baltogiannis et al., 2020; Mates et al., 2020; Markwerth et al., 2021). With the extensive development of molecular genetics, new insights into the SCD causes were sequentially discovered. Pioneering study indicated that rare genetic variants could contribute to important causes of SCD, such as cardiomyopathy, coronary artery disease, and primary arrhythmia syndromes (Khera et al., 2019). Besides, polymorphic variants of *LMNA* and COX10 have been shown to increase the risk of cardiac events (Captur et al., 2018; Yang et al., 2021). Therefore, further genetic analysis seems indispensable to explore more useful genetic markers in unraveling the potential cause of SCD and related diseases (Mates et al., 2018).

The Niemann-Pick type C1 (*NPC1*) protein, a glycoprotein that associates with the membranes of a series of cytoplasmic vesicles (Higgins et al., 1999), regulates cholesterol transport from late endosomes/lysosomes (LE/LY)and plays a vital role in maintaining steady-state levels of cellular cholesterol (Ory 2004; Lamri et al., 2018). Cholesterol plays an essential role in the human body and takes part in various intracellular metabolism (Talamillo et al., 2020). As one of the key modulators of cholesterol metabolism pathway, NPC1 can affect cellular cholesterol homeostasis (Millard et al., 2000), and the imbalanced cholesterol homeostasis is involved in atherosclerosis (Yuan et al., 2012; Du and Yang 2013). Moreover, studies have shown that NPC1 protein-induced apoptosis of macrophages in advanced human atherosclerotic lesions, resulting in plaque instability and disruption (Feng et al., 2003a; Feng et al., 2003b; Yu et al., 2014). The break of atherosclerotic plaque walls can cause acute cardiovascular death (Virmani et al., 2000; Roth et al., 2015). Therefore, considering the essential role in the atherosclerotic progression, one of the major pathological bases of coronary heart disease, dysregulation of NPC1 may be a risk factor that influences SCD development.

Niemann-Pick type C (NPC) patients are characterized by accumulating massive amounts of lipid/cholesterol in the LE/LY and majority of these patients carry mutations in *NPC1* gene (Yu et al., 2014), indicating the genetic regulatory roles underline the dysfunction. Therefore, in this current case-control study, based on systematic genetic variation screening results, an insertion/deletion (indel) polymorphism (rs150703258) downstream of *NPC1* was chosen to investigate its relationship with SCD risk in three distinct geographical Chinese populations. The potential mechanisms of these connections were initially explored.

## 2 Materials and Methods

### 2.1 Ethics Statement

The Ethical Committee of Soochow University has approved this study (approval number: ME81772029). Informed consent was obtained from all healthy blood donors and the victim’s family before the investigation.

### 2.2 Study Populations

A total of 524 controls and 158 SCD cases were included in the study, all of which were ethnic Han Chinese without any genetic relationship. The samples of SCD victims were collected from the following institutions during autopsy cases for medicolegal purpose: Soochow University and Institute of Forensic Sciences (China Ministry of Justice) representing Eastern China (20 cases), Sun Yat-sen University representing Southern China (103 cases) and Guizhou Medical University representing Southwestern China (35 cases). The blood samples were collected for the current study following the inclusive and exclusive criteria described previously (Wang et al., 2017; Zou et al., 2019). In brief, detailed forensic pathological examination was rigorously performed with each SCD case to confirm the coronary heart diseases as the main cause of SCD. No other fatal changes were detected, in addition to varying degrees of coronary atherosclerosis. Age-matched (±5 years) healthy controls were recruited from the same region during the same period as the SCD case, but those with any positive family history of SCD or cardiovascular disorders were excluded. During autopsies on traffic accident victims at the Forensic Medicine Expert Center of Soochow University, a total of 15 human heart tissues were obtained. The myocardium tissues were collected during forensic autopsies and were saved at −80°C until RNA and DNA extraction.

### 2.3 GEO Analysis and Gene Mutation Screening

The GSE41571 and GSE23561 mRNAs profiles from Gene Expression Omnibus (GEO) database were used to analyze the correlation between NPC1 and metabolic syndrome and coronary artery disease (CAD) (Lee et al., 2013; Grayson et al., 2011). Information on all variants of *NPC1* was retrieved from the variation module of the dbSNP database (https://ncbi.nlm.nih.gov/snp/, build 155, access date: June 2021). In sight of uncommon risk alleles with an evolutionary disadvantage, minor allele frequencies >0.15 was established for variant selection. Based on the Genotype-Tissue Expression (GTEx) data, expression quantitative trait loci (eQTL) analysis and Gene Expression Profiling Interactive Analysis (GEPIA) were used to assess the impact of the variants on target gene expression and to measure the expression correlations between genes, respectively (GTEx Consortium 2013). The 1000 Genomes Project data on 445 lymphoblastoid cell lines were used to validate the validity of the results of the analysis (Sudmant et al., 2015; Ferreira et al., 2016). Linkage disequilibrium (LD) analysis was analyzed using R package (Version 4.0.3) (Chan 2018). In addition, genotype data were obtained through the Ensembl Release 105 (December 2021) (http://asia.ensembl.org/Homo_sapiens/Info/Index).

### 2.4 DNA Extraction and Genotyping

Genomic DNA from blood samples and tissues was extracted using Blood DNA Kit or Blood Spots DNA Kit (TIANGEN, Beijing, China). All primers for the amplification were synthesized from Genewiz Company (Suzhou, China). Following the PCR reaction, the genotyping PCR products were detected by 7% non-denaturing PA-gel electrophoresis (PAGE) and visualized through the silver staining method. Genotyping process was performed in a double-blinded way (Zhu et al., 2012). After sample randomization, 20 DNA samples (10 from case and 10 from control) were gel purified and sequenced to confirm the results. In addition, two independent investigators verified approximately 10% (20 case samples and 190 control samples) of total DNA samples to confirm the consistency of genotyping. The following primers were used for genotyping: Forward: 5′-GTG​ATG​TGA​TCT​CTG​CTC​ATT-3′, Reverse: 5′-GGG​CAA​CAA​GTG​GAA​CA-3´.

### 2.5 Quantitative Real-Time PCR Analysis

Total RNAs of each group were extracted from myocardium tissue samples using TRIzol reagent (Invitrogen) and quantified by NanoDrop 2000c Spectrophotometer. The amplification was performed using the SYBR Green dye on the LightCycler real-time PCR system (Roche). The results were calculated using the 2^−ΔΔCT^ algorithm. To exclude potential bias across different conditions, GAPDH was used as the internal reference. The primer sequences were as follows: NPC1- Forward: 5′-GCA​CCT​TTT​ACC​ATC​ACT​CCT​G-3′, NPC1- Reverse: 5′-GGC​CAC​AGA​CAA​TAG​AGC​AGT-3´; GAPDH- Forward: 5′-CTC​TCT​GCT​CCT​CCT​GTT​CGA​C-3′, GAPDH- Reverse: 5′-TGA​GCG​ATG​TGG​CTC​GGC​T-3´.

### 2.6 Western Blot Assay

Total proteins in human myocardial tissues were extracted with RIPA (Radio Immunoprecipitation Assay) lysates. Protein samples were subjected to 8% sodium dodecyl sulfate-polyacrylamide gel electrophoresis (SDS-PAGE), then transferred onto PVDF membranes. After transfer, the membranes were blocked with 5% BSA for 1 h and incubated overnight with the following primary antibodies: Anti-NPC1 (Cat# AB134113, 1:1,000 dilution, Abcam Antibodies); Anti-GAPDH (Cat#AB-M-M001, 1:1,000 dilution, Goodhere Biotechnology). After three TBST rinses, the PVDF membranes were incubated again for 1 h with secondary antibodies (1:1,000 dilution, Biyotime Biotechnology). Finally, the bands were detected with the ECL reagent (ChemiScope, Shanghai).

### 2.7 Cell Cultures

The 293T cell lines were cultured in a 37°C humidified chamber that was supplemented with 5% CO_2_ and Dulbecco’s Dodified Eagle Medium (DMEM) containing 1% penicillin-streptomycin and 10% fetal bovine serum (FBS). The cell lines were characterized by Genetic Testing Biotechnology Corporation (Suzhou, China) using short tandem repeat (STR) markers.

### 2.8 Construction of Reporter Plasmid Vector and Luciferase Reporter Assay

Approximately 350-bp fragments containing rs150703258 located in intergenic regions close to NPC1 gene were synthesized and subcloned into KpnI and SacI sites of pGL3-control vector by Genewiz Company (Suzhou, China). Wild type (pGL3-WT) or mutant type (pGL3-MT) vectors contain the delete allele or insert allele, respectively. The direction and sequence of vectors were verified by direct sequencing.

In one well of a 24-well plate, 1×10^5^ cells were seeded for 24 h and then co-transfected with approximately 400 ng reconstructed vector and 40 ng pRL-SV40 vector (Promega) using jetPRIME^®^ transfection reagent (Polyplus-transfection^®^). As controls, cells transfected with empty pGL3-control vector were used. Luciferase assays were performed at 24 h using a dual luciferase assay system and each group had four duplicate wells and the experiments were repeated at least three times.

### 2.9 Prediction of Transcription Factors

JASPAR (http://jaspar.genereg.net/) was used for transcription factors binding site prediction (Castro-Mondragon et al., 2022). The UCSC genome browser is used for visualization the results. For each TF binding profile in the JASPAR CORE collection, genomes were scanned for matches. PWMScan was used to compute the relative scores and *p*-values (Ambrosini et al., 2018). Shaded boxes represent predicted binding sites for each of the TF profiles in the JASPAR CORE collection. The shading of the boxes indicates the *p*-value of the profile’s match to that position (scaled between 0 and 1,000 scores, where 0 corresponds to a *p*-value of 1 and 1,000 to a *p*-value ≤ 10^−10^).

### 2.10 Statistical Analysis

The SPSS (IBM SPSS Statistics Subscription) and GraphPad Prism 8.4.2 were employed for statistical analysis. Depending on the normality of data, Student’s *t*-test was used to compare continuous data. The odds ratio (OR) and 95% confidence interval (95% CI) were estimated using binary logistic regression to evaluate the association between rs150703258 and SCD risk. The χ^2^ test was used in Hardy-Weinberg equilibrium testing. All tests were two-sided and *p*-values < 0.05 were considered statistically significant.

## 3 Results

### 3.1 Selection of Candidate Variants

**FIGURE 1:**
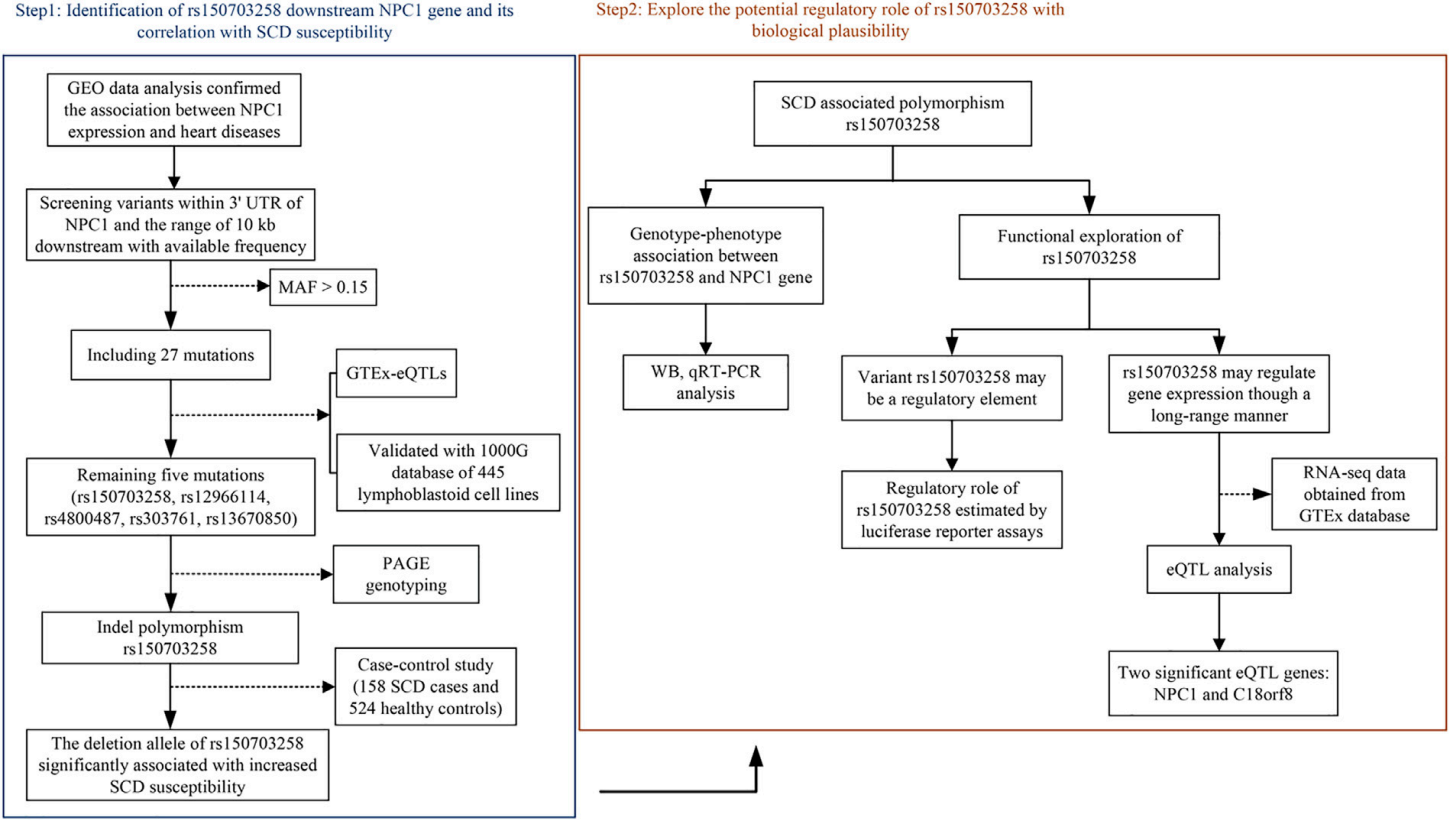
The schematic workflow of the present study. GEO: Gene Expression Omnibus; 3′UTR: 3′untranslated region; MAF: minor allele frequency; GTEx: Genotype-Tissue expression; eQTL: expression quality trait loci; PAGE: polyacrylamide gel electrophoresis; SCD: sudden cardiac death.

**FIGURE 2:**
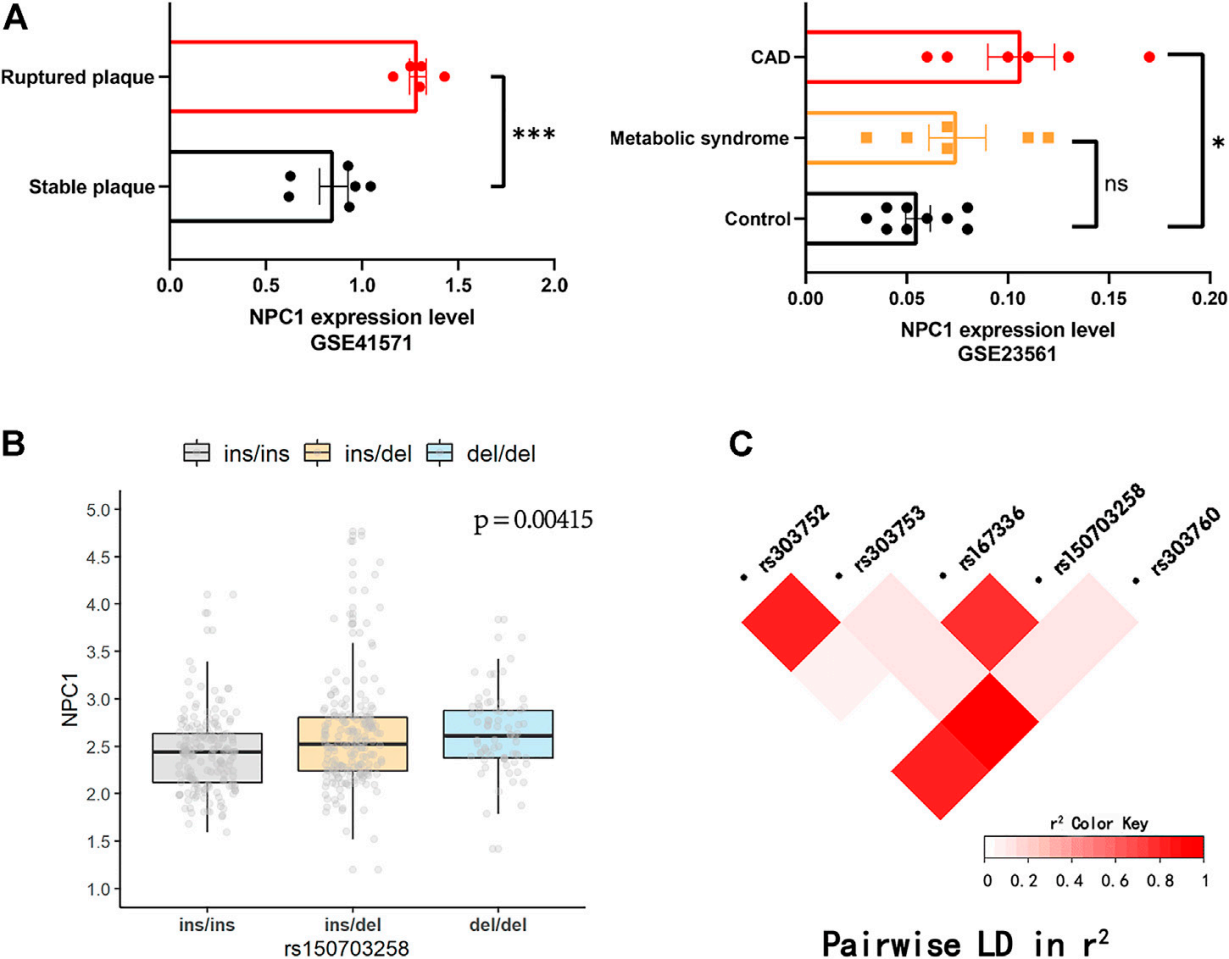
Public database analysis and LD analysis. **(A)** NPC1 was up-regulated in ruptured plaque, metabolic syndrome and coronary artery disease (CAD) tissues. (*p < 0.05, ***p < 0.001). The diversity expression atlases (GSE41571 and GSE23561) were obtained from the Gene Expression Omnibus (GEO) database. **(B)** The validation of the regulatory effect of rs150703258 on expression of NPC1 in 445 lymphoblastoid cell lines in 1000 Genomes Project database. **(C)** Linkage disequilibrium (LD) analysis of rs150703258 and nearby genome-wide association (GWAS) loci.

The workflow of this study was presented in Figure 1. First, the expression level of NPC1 in patients with metabolic syndrome and CAD seems to be higher than that of healthy subjects and a similar trend can be seen from the comparison between the two groups of ruptured plaque and stable plaque (Figure 2A), indicating up-regulation of the NPC1 may lead to an increased risk of cardiac disease. As shown in Supplementary Table S1, we identified 27 variants with a MAF >0.15 within 3′ UTR of *NPC1* and the range of 10-kb downstream region with available frequency. Then, we selected five mutation sites based on eQTL and 1000G data analysis. The four variants were SNPs (rs12966114, rs4800487, rs303761 and rs1367085) and the other one was indel polymorphism (rs150703258). To follow the well-established analysis protocols based on capillary gel electrophoresis combined with PCR-based assays in forensic genetics, indels and other length polymorphisms were given priority in marker selection. Thus, the indel variant rs150703258 was selected as the candidate variant for association investigation. As shown in Figure 2B, rs150703258 polymorphism showed a significant genotype--phenotype association with *NPC1*. Additional LD analysis indicated that rs150703258 and rs167336, which was associated with waist circumference in GWAS analysis, were in the same LD block (Figure 2C).

### 3.2 Correlations Between rs150703258 and Sudden Cardiac Death Susceptibility

**FIGURE 3:**
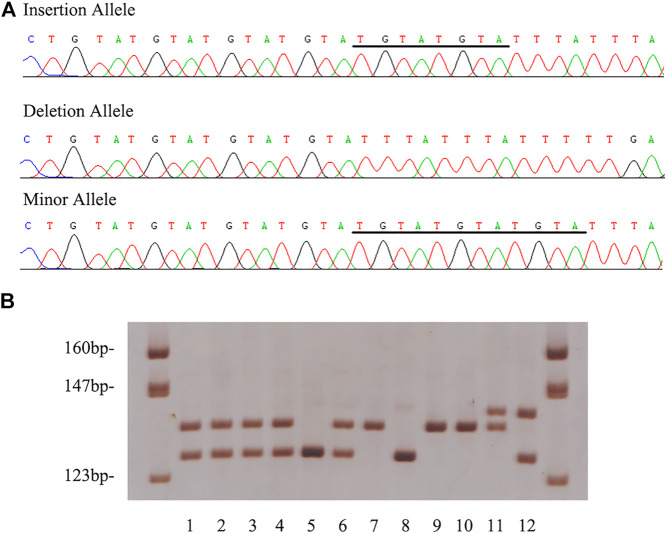
Example output from sequencing and genotyping assays of rs150703258. **(A)** The genotyping outcomes by using 7% non-denaturing polyacrylamide gel electrophoresis (PAGE) and silver staining (lane 7, 9 and 10, ins/ins genotype; lane 1, 2, 3, 4 and 6, ins/del genotype; lane 5 and 8, del/del genotype; lane 11, rare/ins genotype; lane 12, rare/del genotype). **(B)** The sequencing results of insertion, deletion and rare allele in template strands. The underlined bases indicate the “GTA​TGT​AT”/“GTA​TGT​ATG​TAT” insertion in coding strands.

Sample demographic characteristics were given in Table 1. The characteristics of the SCD cases were provided in Supplementary Table S2. Their average age of current SCD cases was around 51-years old and there was a male predilection. Coronary atherosclerosis with various degrees was found in all the SCD cases. Three alleles were found by sequencing in the current cohort. The mutation genotyping results for rs150703258 were shown in Figure 3. A total of five rare 12-bp allele (4-bp longer than the insertion allele) of rs150703258 was discovered in the current cohort. The rare allele ([GTAT]_3_) frequency was calculated to be 0.36%. The observed genotype distributions in the control group have no deviation to the Hardy-Weinberg equilibrium (*p* > 0.05).

**TABLE 1:** Clinical characteristics of SCD cases and controls.

Characteristic	Group	
	Case	Control
No. of individuals	158	524
Sex, No		
Male	139	458
Female	19	66
Age, mean ± SD (range)		
Overall	51.23 ± 14.02 (18–92)	49.82 ± 13.39 (16–96)
Males	50.66 ± 12.96 (23–87)	49.23 ± 12.37 (16–90)
Females	55.42 ± 19.63 (18–92)	53.91 ± 18.54 (22–96)
Events at sudden death (SD)		
Physical activity	37	
Stress	52	
Sleep	13	
Nonspecific	56	
Symptoms before SD		
None	98	
Others	60	
Megalothymus		
Positive	2	
Negative	156	

Genotypic and allelic frequencies of rs150703258 and association analysis were shown in Table 2. Compared with the ins/ins genotype, an increased SCD risk was observed for individuals carrying ins/del genotype (adjusted OR: 1.663, 95% CI: 1.11–2.50, *p* = 1.35 × $10^{-2}$ ). Similar trend was also found in the dominant model (adjusted OR: 1.659, 95% CI: 1.13–2.45, *p* = 1.01 × $10^{-2}$ ). The 8-bp deletion allele could contribute to a 32.9% increased risk of SCD (OR: 1.329, 95% CI: 1.03–1.72, *p* = 2.89 × $10^{-2}$ ). Together, our results indicated that deletion allele of rs150703258 was associated with SCD susceptibility in Chinese populations. In addition, as shown in Supplementary Figure S1, body mass index (BMI) analysis revealed that the genotype of rs150703258 was also associated with BMI (mean BMI = 23.34, 24.55 and 25.07 for ins/ins, ins/del and del/del genotype, respectively).

**TABLE 2:** Associations between rs150703258 and sudden cardiac death susceptibility in case control sets recruited during 2012–2019.

Genetic Model	Genotype	Cases	(%)	Control	(%)	OR (95% C.I.)^a^	*p* Value
Codominant model	ins/ins	45	28.48	208	39.69	1.00 (Reference)	
	ins/del	86	54.43	239	45.61	1.663 (1.11–2.50)	0.0135
	del/del	26	16.46	73	13.93	1.646 (0.95–2.86)	0.0748
	*P*_trend_						0.0261
	rare/ins	1	0.63	3	0.57	—	—
	rare/del	0	0.00	1	0.19	—	—
Dominant model	ins/ins	45	28.66	208	40.00	1.00 (Reference)	
	ins/del + del/del	112	71.34	312	60.00	1.659 (1.13–2.45)	0.0101
Additive model	ins allele	177	56.01	658	62.79	1.00 (Reference)	
	del allele	138	43.67	386	36.83	1.329 (1.03–1.72)	0.0289
	rare allele	1	0.32	4	0.38	—	—

a Adjusted by age and sex. CI, confidence interval; OR, odds ratio.

### Correlations Between rs150703258 Genotype and NPC1 Expression

**FIGURE 4:**
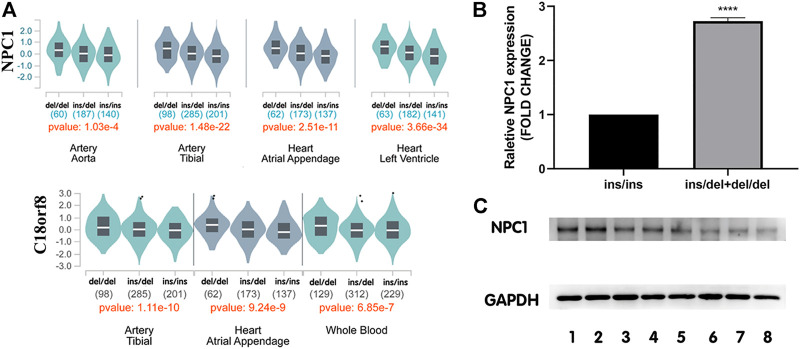
Expression levels of NPC1 in human tissues among different genotypes. **(A)** Genotype-expression analysis between rs150703258 and mRNA expression in human heart, arteries and whole blood samples in GTEx database. **(B)** The mRNA level of NPC1 in human myocardium samples showed that in tissues with ins/del and del/del genotype was 2.73-fold higher than that in samples with ins/ins genotype. (****p < 0.0001). ins/ins, *N* = 6, ins/del and del/del, N = 9. **(C)** Western blot assay analysis of NPC1 at protein level. Lanes 1–2 represent del/del genotype, and lanes 3–5 represent ins/del genotype, and lanes 6-8 represent ins/ins genotype.

The GTEx database was used for eQTL analysis to identify target genes potentially regulated by rs150703258. Our eQTL analysis observed that the rs150703258 variant is highly related to NPC1 and C18orf8 expression levels. In human arteries and heart tissues, both genes have been shown to be critical eQTL genes (Figure 4A).

To further investigate the genotype-phenotype correlations between rs150703258 and *NPC1* gene, *NPC1* expression at mRNA level and protein level was validated by quantitative real-time PCR and western blot in human myocardium tissue samples, respectively. The relative mRNA expression level of *NPC1* in samples with ins/del and del/del genotype was significantly higher than those with ins/ins genotype (Figure 4B) with a fold change = 2.73, p < 0.0001. At protein level, the same trend was also observed in western blot analysis (Figure 4C). The results showed that the deletion allele genotype of rs150703258 was significantly associated with increased gene expression, indicating its potential regulatory role.

### Correlations Between NPC1 and C18orf8 Expression

**FIGURE 5:**
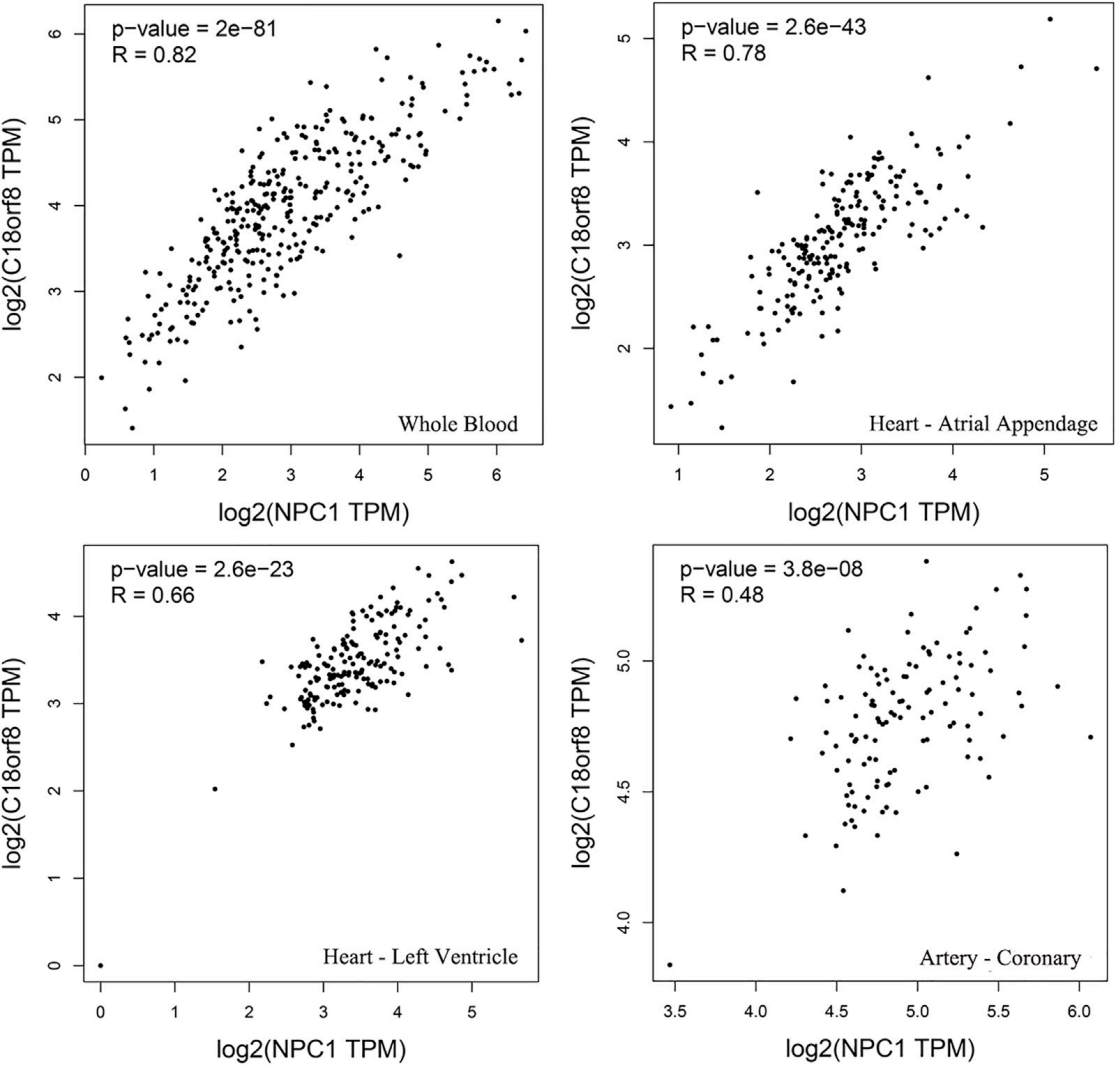
Gene expression profiling interactive analysis. The expression of the two genes indicates a significant correlation in human whole blood, atrial appendage, left ventricle and coronary tissues.

Based on Gene Expression Profiling Interactive Analysis, the expression of NPC1 and C18orf8 presents a significant correlation in human blood, arteries and heart tissues (Figure 5). The similar expression patterns of NPC1 and C18orf8 indicate that the two genes may be co-regulated (*R* = 0.82 in whole blood).

### The Regulatory Role of rs150703258 on Gene Transcription Activity

**FIGURE 6:**
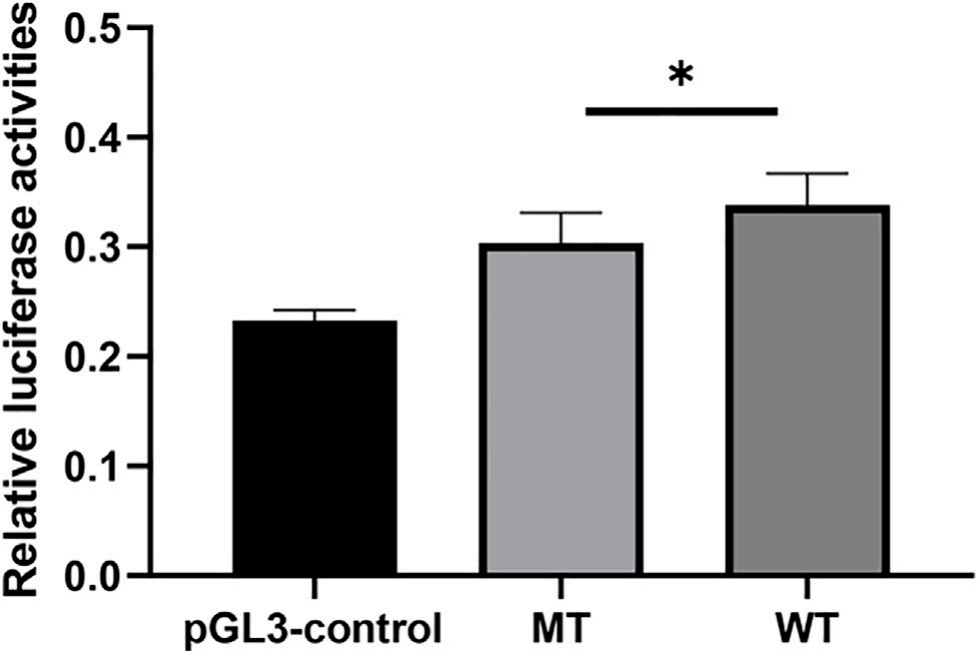
The effect of rs150703258 on gene transcriptional activity. The relative firefly luciferase activities were compared between insertion construct group (pGL3-MT) and deletion construct group (pGL3-WT) in 293T cell lines. Cells transfected with pGL3-WT exhibited a significantly higher luciferase activity as compared with cells transfected with pGL3-MT (*p < 0.05).

**FIGURE 7:**
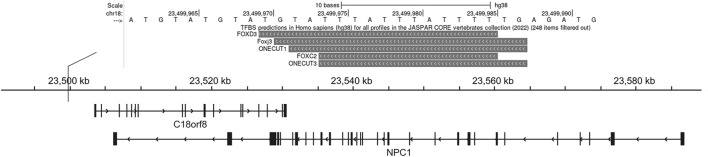
Transcription factor prediction. This UCSC Genome Browser track hub represents genome-wide predicted binding sites for transcription factor binding profiles in the JASPAR database CORE collection, this view only showed predicted binding sites with scores above 500, which indicated the *p*-value of the TF match to indel position was less than 0.00001.

Using pGL3-control vector containing insertion or deletion allele of rs150703258 variant, we performed luciferase reporter assays to confirm whether the indel variant affects transcription activity. As shown in Figure 6, there was a significant difference in luciferase activities between the groups transfected with vectors carrying insertion allele (pGL3-MT) and those carrying deletion allele (pGL3-WT). Luciferase activity in the pGL3-WT transfection group was higher than that in the pGL3-MT transfection group, indicating that rs150703258 showed regulatory properties on gene transcription in this study. Additionally, we surmised that the deletion allele of rs150703258 may regulate gene expression by recruiting transcription factors. In *silico* analysis of this region showed that the rs150703258 deletion variant may create transcription factor binding sites (Figure 7).

## 4 Discussion

Accurate diagnosis and predictive genetic testing of SCD is still challenging in forensic and clinical community. Exploration more useful and valuable genetic markers is indispensable in forensic molecular autopsy of SCD victims and genetic counselling of their relatives. Based on the mutation screening and subsequent case-control study, we successfully identified the novel 8-bp indel mutation (rs150703258) within downstream area of *NPC1* gene associated with susceptibility to SCD in Chinese populations. Further genotype-phenotype correlation analysis based on eQTL and our *in vivo* data revealed a significant association between rs150703258 and NPC1 expression, indicating the potential regulatory roles of the novel indel. In addition, a rare 12-bp variation (minor allele frequency <1%) of rs150703258 was discovered in the current cohort. The polymorphic region seems like a kind of simple “GTAT” repeat expansion, reflecting the short tandem repeat (STR) characteristics of rs150703258. Collectively, this indel may serve as a useful genetic marker for SCD risk stratification as well as molecular diagnosis.

Cholesterol is one of the main reasons that lead to atherosclerosis, which is the pathological basis of coronary heart disease (CHD) (Roberts 2017). Notably, CHD still underlies the majority of SCD. Foam cell formation due to the excessive accumulation of cholesterol by macrophages is a major hallmark of early-stage atherosclerotic lesions (Yu et al., 2013). During the further progression of the atherosclerotic disease, the lesion stability also seems to be affected by macrophages. (Pennings et al., 2006). As the key partner in promoting intracellular trafficking of cholesterol, NPC1 has been demonstrated to become a vital role in the cardiovascular system. In macrophage cell lines, a high level of NPC1 and deletion of NPC1 is primarily responsible for the accelerated atherosclerosis (Tomkin 2008). There are several genetic mutations in the *NPC1* gene associated with cardiovascular disease (CVD) (Afzali et al., 2017), CHD (Ma et al., 2010), overweight, obesity and morbid obesity (Sandholt et al., 2011). For instance, research headed by Afzali reported that the rs1805081 (+644A > G) of *NPC1* was associated with reduced CVD risk in the Iranian population (Afzali et al., 2017). Ma and others (Ma et al., 2010) found that *NPC1* variants increased the incidence of CHD in the Chinese population. Some *in vivo* and *in vitro* studies also provided evidences that *NPC1* gene take part in the development and progression of atherosclerosis. For example, a previous study noted that intracellular cholesterol trafficking defect of the nonsense mutant *NPC1* gene predisposes to increased atherothrombosis, lesion formation, and medial degradation (Welch et al., 2007). In addition to that, *NPC1* deficient mice can reconstitute with LDL receptor and accelerated aortic atherosclerosis was observed in the *NPC1* ^−/−^ macrophages (Zhang et al., 2008).Furthermore, NPC1 repression was found to invoke lipid accumulation in human macrophages exposed to environmental aryl hydrocarbons (Podechard et al., 2009). On the other hand, Feng et al. have demonstrated that the atherosclerotic lesions of the *NPC1* ^+/−^;*ApoE* ^−/-^ mice showed less necrosis and more cells than lesions in *NPC1* ^+/+^;*ApoE* ^−/-^ mice, the latter showed extensive acellular areas (Feng et al., 2003b). Based on these data, it appears that *NPC1* gene variants and the imbalance of NPC1 could play an essential role in atherosclerosis and CVD, which are the two significant etiologies of SCD.

*NPC1* plays a crucial role in the development of atherosclerosis. Nevertheless, it is previously reported that promoter DNA methylation could reduce NPC1 expression and contribute to atherosclerosis progression (Afzali et al., 2013). Conversely, LXR agonist has been shown to inhibit atherosclerotic lesion formation and induce regression of established lesions in *ApoE* ^−/-^ mice through upregulation of NPC1 expression (Dai et al., 2008). These conflicted with the above observations which characterize NPC1 as an atherosclerosis promoting factor (Feng et al., 2003b). It may be interpreted by different influences of NPC1 on different stages of coronary atherosclerosis. For example, in advanced stage of atherosclerosis, overexpression of NPC1 could reverse plaque vulnerability and increase the risk of SCD.

Further, we found that rs150703258 is located upstream of *C18orf8* and downstream of *NPC1*. Importantly, both genes showed a strong correlation in expression. It is interesting to note that C18orf8 as a new subunit of the CCZ1-MON1 RAB7 guanine exchange factor (GEF) that positively regulates the recruitment of RAB7 to LE/autophagosomes during endocytic trafficking (Vaites et al., 2017). Therefore, researchers refer to C18orf8 as RMC1 (regulator of MON1-CCZ1). Further evidence from van den Boomen et al.’s studies confirmed that C18orf8 indeed regulates the expression of the NPC1 gene, and the MON1-CCZ1-C18orf8（MCC）complex plays a critical role in NPC1-dependent lysosomal cholesterol export (van den Boomen et al., 2020). Nevertheless, the relative mechanism remains at present unclear. Moreover, our analysis revealed that this mutation is enriched with transcription factor binding sites. Taking these factors into account, the deletion allele of rs150703258 may recruit transcription factors or change the expression level of C18orf8 and thus affect NPC1 expression. However, whether these are triggering variability in NPC1 expression remains to be explored. The function of rs150703258 in NPC1-dependent lysosomal cholesterol export will be further understood in future studies.

Based on further LD analysis, we found that rs150703258 was in one complete LD block with rs167336, a significant GWAS SNP associated with waist circumference phenotype, one of the cardiometabolic risk factors of metabolic syndrome (Tachmazidou et al., 2017; Lind 2020). The results indicated that this specific genomic region may harbor real causative variants for cardiometabolic risk. It also addressed the possibility that both variants may be functional which needed further molecular mechanism investigation.

However, the current study has some limitations that should be addressed. Firstly, the current cohort consists of a relatively small sample size. Studies incorporating larger sample sizes are required to validate the risk of rs150703258 on SCD susceptibility. Secondly, further research is required to understand better the molecular mechanisms that link rs150703258 to NPC1 expression and SCD development.

As the golden standard for SCD diagnosis, forensic autopsy of SCD cases is an indispensable step in thorough investigating the underlying pathology, emphasizing its the essential roles in SCD diagnosis and prophylactics. SCD can be caused by different types of conditions, including atherosclerosis, arrhythmias, cardiomyopathy and channel disorders. It was worthy of note that almost all the SCD cases in the current study suffered from moderate to severe coronary atherosclerosis, supporting atherosclerosis can be one of the pathophysiological substrates for SCD risk. Similarly, NPC1 has been shown to be associated with obesity (Lamri et al., 2018). Our results also revealed that the genotype of rs150703258 was associated with BMI, indicating the genetic contributing roles of NPC1 to obesity. The complex spectrum of these comorbidities may influence the clinical manifestations and should be taken into account in the diagnosis and prevention of individuals with SCD risk.

## 5 Conclusion

In conclusion, our preliminary findings indicate that rs150703258 downstream of *NPC1* seems to be a contributor to SCD susceptibility among Chinese populations. Thus, the novel indel rs150703258 may be a potential marker for risk stratification and molecular diagnosis of SCD.

## Data Availability

The original contributions presented in the study are included in the article/Supplementary Material, further inquiries can be directed to the corresponding authors.
