# Supplementary figures and images for: Novel Indel Variation of NPC1 Gene Associates With Risk of Sudden Cardiac Death
Source: Front Genet. 2022 Apr 11;13:869859. doi: 10.3389/fgene.2022.869859 (PMC9035640; doi:10.3389/fgene.2022.869859)

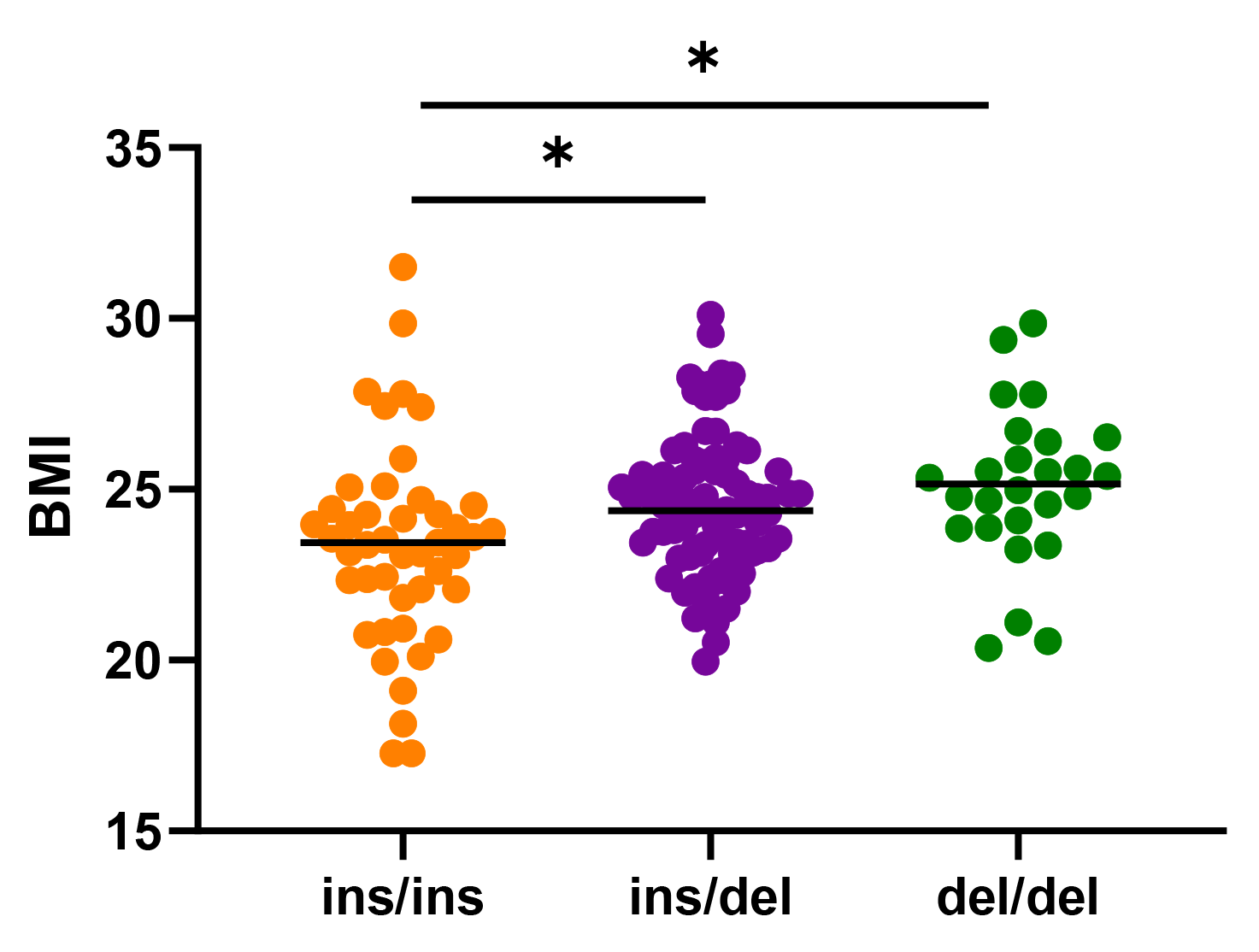

Supplement: Supplementary file 2 [file Image1.TIF]
